# Obituary

**Published:** 1868-08

**Authors:** 


### OBITUARY.

It is our sad duty to record the death of Dr. GAM'L JACKSON, of Winona, Minn., and formerly of this city, which ocurred on Monday morning, May 31.

For some time past he has been succumbing to that dreaded disease consumption, so that his death was not unlooked for.

Dr. JACKSON was born at Steubenville, Ohio, and was thirty-four years of age at the time of his decease. He removed to Winona about five years ago and entered upon the practice of Dentistry; always sustaining a reputation as a high-minded and honorable member of the profession. He was a man of more than ordinary culture and genius, and those who know him best, can attest to the purity of mind and heart which he evinced in his intercourse with his friends. He leaves a wife and child, who have the sympathy of warm friends in their bereavement.

In his death the profession has lost a very promising member.

